# Integrated Analysis of Gut Microbiome and Lipid Metabolism in Mice Infected with Carbapenem-Resistant *Enterobacteriaceae*

**DOI:** 10.3390/metabo12100892

**Published:** 2022-09-22

**Authors:** Ning Zhang, Yuanyuan Peng, Linjing Zhao, Peng He, Jiamin Zhu, Yumin Liu, Xijian Liu, Xiaohui Liu, Guoying Deng, Zhong Zhang, Meiqing Feng

**Affiliations:** 1 School of Chemistry and Chemical Engineering, Shanghai Engineering Research Center for Pharmaceutical Intelligent Equipment, Shanghai Frontiers Science Research Center for Druggability of Cardiovascular Noncoding RNA, Shanghai University of Engineering Science, Shanghai 201620, China; 2 Minhang Hospital & School of Pharmacy, Fudan University, Shanghai 200433, China; 3 Shanghai Engineering Research Center of Immunotherapeutic, Shanghai 201203, China; 4 Instrumental Analysis Centre, Shanghai Jiao Tong University, Shanghai 200240, China; 5 Trauma Center, Shanghai General Hospital, Shanghai Jiao Tong University School of Medicine, Shanghai 201620, China; 6 Nursing Department, Shanghai General Hospital, Shanghai Jiao Tong University School of Medicine, Shanghai 201620, China

**Keywords:** carbapenem-resistant *Escherichia coli*, lipid disturbance, metagenomics, metabolomics, lipidomics, association analysis, *Erysipelotrichaceae* family

## Abstract

The disturbance in gut microbiota composition and metabolism has been implicated in the process of pathogenic bacteria infection. However, the characteristics of the microbiota and the metabolic interaction of commensals–host during pathogen invasion remain more than vague. In this study, the potential associations of gut microbes with disturbed lipid metabolism in mice upon carbapenem-resistant *Escherichia coli* (CRE) infection were explored by the biochemical and multi-omics approaches including metagenomics, metabolomics and lipidomics, and then the key metabolites–reaction–enzyme–gene interaction network was constructed. Results showed that intestinal *Erysipelotrichaceae* family was strongly associated with the hepatic total cholesterol and HDL-cholesterol, as well as a few sera and fecal metabolites involved in lipid metabolism such as 24, 25-dihydrolanosterol. A high-coverage lipidomic analysis further demonstrated that a total of 529 lipid molecules was significantly enriched and 520 were depleted in the liver of mice infected with CRE. Among them, 35 lipid species showed high correlations (|r| > 0.8 and *p* < 0.05) with the *Erysipelotrichaceae* family, including phosphatidylglycerol (42:2), phosphatidylglycerol (42:3), phosphatidylglycerol (38:5), phosphatidylcholine (42:4), ceramide (d17:1/16:0), ceramide (d18:1/16:0) and diacylglycerol (20:2), with correlation coefficients higher than 0.9. In conclusion, the systematic multi-omics study improved the understanding of the complicated connection between the microbiota and the host during pathogen invasion, which thereby is expected to lead to the future discovery and establishment of novel control strategies for CRE infection.

## 1. Introduction

Carbapenem-resistant *Escherichia coli* (CRE) represent a severe public health problem [1,2,3]. Infections caused by CRE, such as complicated urinary tract infections, bloodstream infections and pneumonia, are alarming in the clinical setting which are often associated with high mortality [4,5]. Therefore, studies on the pathological mechanism and treatment strategy against CRE infection are extremely urgent.

Gut microbiome, which is involved in the regulation of multiple metabolic pathways of the host [6], could assist in the development of new strategies for infectious diseases [7,8]. One of the major functions of the gut microbiome is to prevent the colonization of pathogens and overgrowth of indigenous pathogens [9]. Dysbiosis of gut microbiota could lead to pathologic immune responses, reducing the integrity and function of the intestinal barrier and accelerating the infection process [10]. There has been increasing evidence demonstrating the remarkable impact of gut microbiome on determining the susceptibility to CRE carrying and eventual infection [4,11,12]. A similar finding has been made in our previous study [13]. The basic view of bacterial pathogenesis during infection is the ability of an invader to overcome innate host defenses and the barrier of the resident microbiota [14]. However, the complex interplay between the pathogen, the microbiota and the host, as well as their metabolic characteristics, remains largely unknown so far.

Lipidomics is becoming an increasingly powerful tool for systems biology, which greatly expands the fields of traditional repertoire [15,16,17]. The comprehensive study of lipidome based on mass spectrum analysis has been representatively applied in medical microbiology such as infection diagnoses [18]. The metabolically active gut microbial community has a profound effect on the absorption, digestion, metabolism and excretion of lipids [19]. Previous studies have suggested that the gut microbiome plays a particularly important role in the regulation of host cholesterol and sphingolipid homeostasis [20]. The microbiota-modified triglyceride and phosphatidylcholine species in the liver [21] and the excessive production of short-chain fatty acids by intestinal bacteria contributed to the accumulation of lipids in the liver [22].

To the best of our knowledge, there is no publication to investigate the associations of altered gut microbiome and lipid metabolism that occur during CRE infection. In this work, the biochemical and multi-omics technologies, including metagenomics, metabolomics and lipidomics, were used to decipher a key gut microbe which had high association with the lipid disturbance for CRE invasion, and a few targets potentially responsible were identified for future study.

## 2. Materials and Methods

### 2.1. Chemicals and Bacteria

Methanol, chloroform, acetonitrile, isopropyl alcohol and dichloromethane of HPLC grade were purchased from Thermo-Fisher Scientific (Fair Lawn, NJ, USA). Butylhydroxytoluene (BHT) was purchased from ANPEL Laboratory Technologies (Shanghai) Inc. Ultrapure water was freshly prepared by a Milli-Q reference system (Millipore, Bedford, MA, USA). The clinical isolate of CRE (No. 1864) from human rectal swabs was obtained from the Huashan Hospital, Fudan University, China.

### 2.2. Animals

The experimental protocol was approved by the animal ethics committee of the school of pharmacy, Fudan University. Six-week-old female ICR mice (16–18 g) were obtained from SLAC Laboratory Animal Co., Ltd. (Shanghai, China). All mice were fed in a barrier system with temperature (24 ± 2 °C), humidity (60 ± 10%) and 12/12 h light/dark cycle, and provided with certified standard rat chow and tap water ad libitum. After one week of acclimatization, mice were randomly assigned into two groups, including the normal control group (NC, n = 8) and the CRE-infected group (CRE, n = 10). Mice in CRE group received a single intraperitoneal injection of CRE in saline (1 × 10^8^ CFU/mL, 200 μL), while mice in NC group were injected intraperitoneally with an equal amount of saline. After 24 h of infection, including the final 12 h of fasting, mice were sacrificed by cervical dislocation, and the samples of feces, liver and abdominal adipose tissue were collected. All samples were promptly frozen in liquid nitrogen, stored at −80 °C, pending for biochemical, metagenomic, metabolomics and lipidomic analyses.

### 2.3. Biochemical Analyses

Hepatic lipids were extracted by the previously published method [23]. Briefly, the liver tissues were homogenized with chloroform/methanol (2/1, *v*/*v*) to a final volume 20 times that of the tissue sample and followed by a series of dispersion, agitation and centrifugation steps. The hepatic levels of triglyceride (TG), total cholesterol (TC), high-density lipoprotein cholesterol (HDL-c) and low-density lipoprotein cholesterol (LDL-c) were measured by ELISA kits (Huili Biotech, Changchun, China) and a Chemray 240 fully automatic biochemical analyzer (Rayto, Shenzhen, China).

### 2.4. Metagenomics

The feces samples were used for intestinal microbiota analysis using 16S rDNA sequencing, as described in detail in our previous paper [13].

### 2.5. Metabolomics

The serum and fecal samples were pretreated, and the derivatives were detected and analyzed by Agilent 7890B gas chromatograph system coupled to a Leco Pegasus time-of-flight mass spectrometer (GC-TOFMS). See the previous report for more details [13].

### 2.6. Lipidomics

#### 2.6.1. Sample Preparation

Lipids in live samples were extracted by the method reported by Bligh and Dyer [24]. Briefly, 300 µL mixture of methanol/acetonitrile/water (2/2/1 by volume) along with 0.1% BHT was added in 50 mg of liver tissue and homogenized for 2 min. Then, vortexed for 30 s and centrifuged at 12,000 rpm, 4 °C for 5 min. The supernatant was transferred to a new tube. The extraction was repeated twice. Then, 560 µL of chloroform were added, vortexed for 30 s and centrifuged at 12,000 rpm, 4 °C for 5 min. The under layer organic phase was transferred to auto-sample vials and concentrated to dryness. The extract was reconstituted using the 100 μL of dichloromethane/isopropyl alcohol/methanol solution (1/1/2 by volume). Quality control (QC) sample was prepared by pooling some of the reconstituted solutions of each sample; then analyzed by the same procedure.

#### 2.6.2. LC-MS/MS Method for Lipid Analysis

An ACQUITY-ultraperformance liquid chromatography (UPLC) system (Waters Corporation, Milford, CT, USA) was used for the separation on a Waters BEH C18 column (100 × 2.1 mm) with 1.7-micrometer particles at 55 °C. The mobile phase consisted of acetonitrile/water (60:40) with 10 mM ammonium formate and 0.1% formic acid (solvent A), and isopropanol/acetonitrile (90:10) with 10 mM ammonium formate and 0.1% formic acid (solvent B), with a flow rate of 0.4 mL/min. The gradient was 95/5~0/100 in 17 min. The injection volumes were 1 μL for ESI (+) mode and 4 μL for ESI (−) mode, respectively.

The mass spectrometric data were collected using a Thermo Scientific Q-Exactive Plus mass spectrometry (QE-MS). ESI was used as the ionization source, and the analysis was carried out in both positive mode and negative mode. The Scan mode was set at DDA mode, 1 full scan followed by 6 MS/MS scans. Collision energy was NEC 15, 30, 45 to fragment the ions. Nitrogen (99.999%) was used as collision-induced dissociation gas. The other conditions for the MS were as follows: capillary temperature of 320 °C, the spray voltage of 3.8 kV in positive mode and 3.0 kV in negative mode, S-Lens RF Level of 50 V and the scan range of 150 to 2000 amu.

#### 2.6.3. Lipids Identification

Data were processed by XCalibur software (Thermo Fisher Scientific, San Jose, CA, USA) for peak picking, alignment and normalization to produce peak intensities for retention time (RT) and m/z data pairs. Compounds were identified based on accurate mass; fragments in MS/MS using LipidSearch software.

### 2.7. Statistics

The metabolome and lipidome data were imported into the SIMCA 14.1 software (Umetrics, Umeå, Sweden) for supervised orthogonal partial least squares discriminant analysis (OPLS-DA). The discriminatory metabolites in serum and feces, as well as the lipid species in live, were identified by the multivariate and univariate statistical analyses, with the criteria of VIP > 1 in OPLS-DA, *p* < 0.05 using a two-tailed paired Student’s *t*-test and fold change >1.2 or <0.8. False discovery rate (FDR) value was obtained to reduce the risk of a false positive by the adjusted *p* value using the Benjamini and Hochberg method [25]. All the bar plots in this study were generated with GraphPad Prism (version 9.0, GraphPad Software, San Diego, CA, USA). Heatmap was performed using Euclidean Dist algorithm by TBtools software. Cross-omics association study was performed by Spearman correlation analysis and presented by SPSS 26.0 (IBM Corp., Armonk, NY, USA) and R 4.0.5 software until August 2022.

### 2.8. Network Analysis and Potential Targets Prediction

The lipids data were imported into Cytoscape 3.7.1 (https://cytoscape.org/, accessed on 26 August 2022) to visualize the associations of lipids and their co-regulating characteristics. The metabolites–reaction–enzyme–gene network was constructed by Metscape. The genes associated with the significantly changed lipids, which were considered as the potentially responsible targets for CRE infection, were uploaded to STRING (https://cn.string-db.org/, accessed on 26 August 2022) to construct protein–protein interactive network.

## 3. Results

### 3.1. Hepatic Lipid Profile Analysis

**Figure 1:**
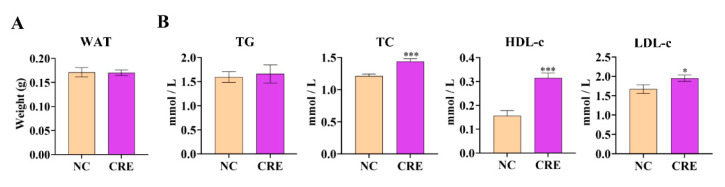
(**A**) Abdominal white adipose tissue (WAT) weights. (**B**) Hepatic lipid profile of TG, TC, HDL-c and LDL-c in mice. Data are presented as mean ± SEM. *p* values were determined using an unpaired, two-tailed Student’s *t*-test. * *p* < 0.05, *** *p* < 0.001.

The 24 h infection of CRE had no phenotypic effect with the comparable body weight and white adipose tissue weight relative to controls (Figure 1A). Strikingly, the hepatic levels of TG, TC, HDL-c and LDL-c were all increased in CRE-infected mice compared with the control group (Figure 1B). The increases in TC and LDL-c were statistically significant (*p* < 0.001 or *p* < 0.05), which suggested that lipid disturbance had occurred in the early stage of infection of CRE.

### 3.2. Gut Microbiota Composition Analysis

**Figure 2:**
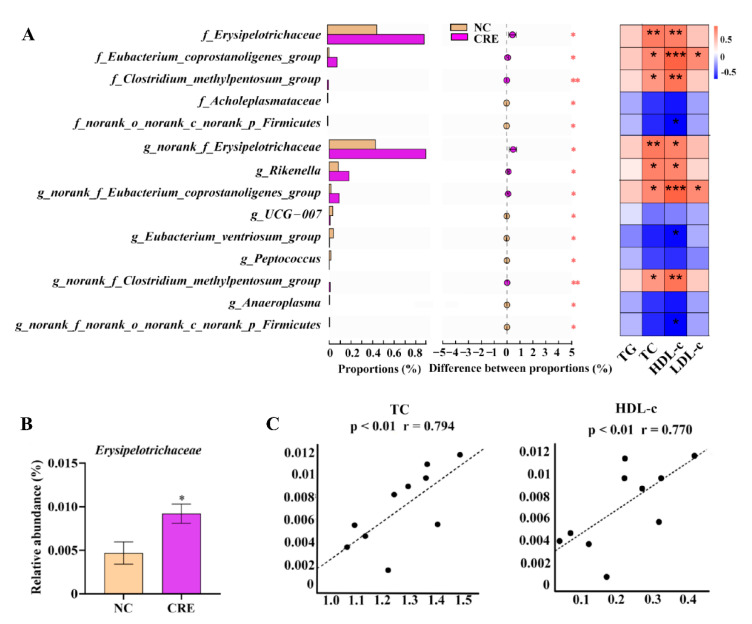
(**A**) Significantly differential gut microbes at the family and genus levels in CRE infection group compared with controls (**left**), and their correlations with the hepatic lipid profile of TG, TC, HDL-c and LDL-c (**right**). (**B**) Relative abundance of intestinal *Erysipelotrichaceae* at the family level; (**C**) correlation plot of *Erysipelotrichaceae* family with hepatic levels pf TC and HDL-c. Vertical coordinates are the relative abundance values of *Erysipelotrichaceae* (%), and the horizontal coordinates are the TC or HDL-c levels (mmol/L), respectively. Data are presented as mean ± SEM. *p* values were determined using an unpaired, two-tailed Student’s *t*-test. * *p* < 0.05, ** *p* < 0.01, *** *p* < 0.001.

The 16S rDNA sequencing technology was employed to investigate the CRE-related alterations of gut microbiome in family and genus levels, which were calculated by the summations of all the OTUs of the corresponding family and genus, respectively. A total of five families and nine genera were significantly different in the CRE group relative to controls through LEfSe analysis, as shown in Figure 2A. Three families including *Erysipelotrichaceae*, *Eubacterium_coprostanoligenes* and *Clostridium methylpentosum* were positively correlated with hepatic lipids. Among them, the *Erysipelotrichaceae* family had the highest relative abundance. Compared with the controls, the relative abundance of *Erysipelotrichaceae* in the CRE group increased significantly (Figure 2B). The strong correlation of the intestinal *Erysipelotrichaceae* family was found with the hepatic levels of TC (r = 0.794, *p* < 0.01) and HDL-c (r = 0.770, *p* < 0.01) (Figure 2C).

### 3.3. Serum and Feces Metabolomics Analysis

**Figure 3:**
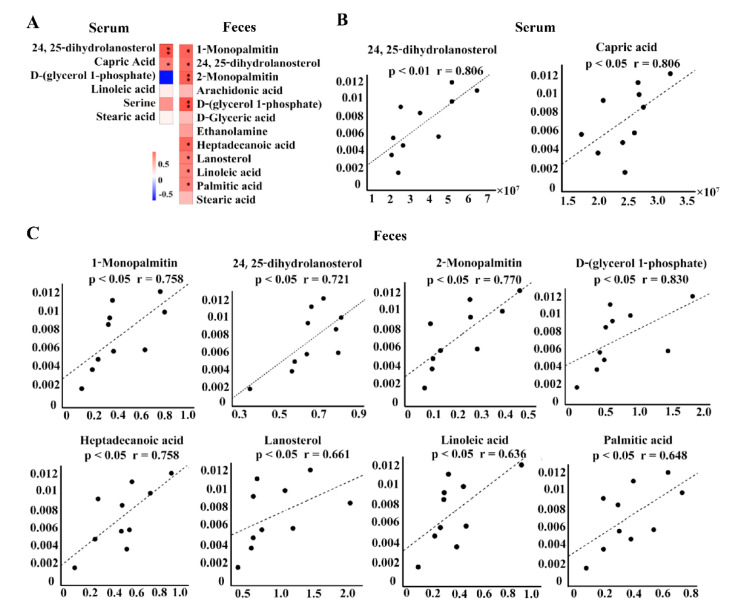
Spearman’s correlation results (**A**) and the corresponding scatter plots (**B**,**C**) showing the associations of intestinal *Erysipelotrichaceae* family with the metabolites related to lipid metabolism in serum and feces. Vertical coordinates are the relative abundance of *Erysipelotrichaceae* (%), and horizontal coordinates are the intensities of metabolites after correction (**C**). * *p* < 0.05, ** *p* < 0.01.

Untargeted metabolomics analysis showed that a total of 74 metabolites in sera and 129 in feces had a significantly different response to CRE infection (Tables S1 and S2). Among them, the six metabolites in sera and twelve in feces were involved in the lipid metabolism. We further investigated the association of the alteration of the intestinal *Erysipeltrichaceae* family with these metabolites. As shown in Figure 3, the abundance of intestinal *Erysipeltrichaceae* family had significantly positive correlation with the serum levels of 24,25-dihydrolanosterol (*p* < 0.01) and capric acid (*p* < 0.05), and the fecal levels of 2-monopalmitin (*p* < 0.01), glycerol 1-phosphate (*p* < 0.01), 1-monopalmitin (*p* < 0.01), 24,25-dihydrolanosterol (*p* < 0.01), heptadecanoic acid (*p* < 0.05), lanosterol (*p* < 0.05), linoleic acid (*p* < 0.05) and palmitic acid (*p* < 0.05). In particular, the changes in 24,25-dihydrolanosterol in both serum and feces were closely related with intestinal *Erysipelotrichaceae* family, which is a lipid metabolite produced by commensals–host interaction.

### 3.4. Hepatic Lipidomes Analysis

To elucidate the lipid characters of CRE infection, we used the ultraperformance liquid chromatography-Q Exactive plus mass spectrometer (UPLC-QEMS) to detect the hepatic lipidome and analyzed the differences.

**Figure 4:**
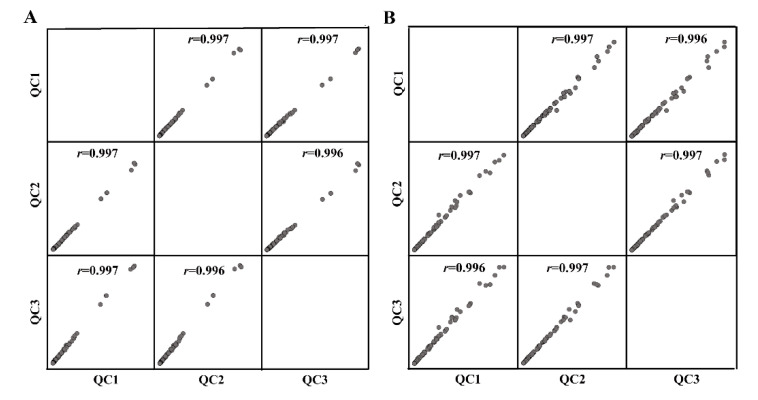
Correlation analysis of quality control (QC) samples in positive ion modes (**A**) and negative ion modes (**B**), respectively, by UPLC/QEMS-based lipidomics.

Data quality was assessed by three QC samples. Correlation analysis showed good reproducibility between QCs, with the correlation coefficient value of more than 0.99 (Figure 4), showing a stable analysis system of lipidomics.

**Figure 5:**
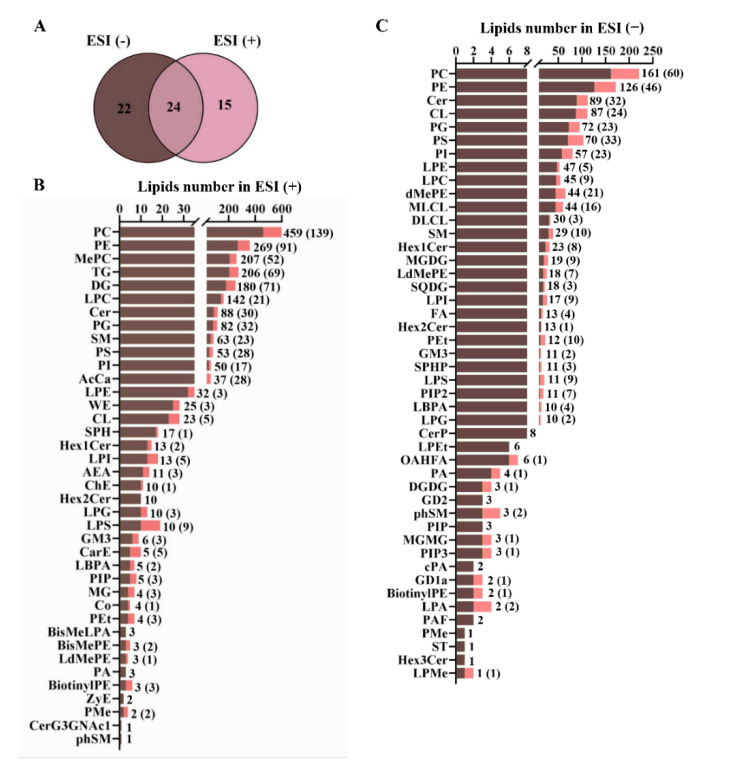
(**A**) Venn diagrams showing the quantity of lipid subclasses detected in positive and negative ion modes. The numbers of identified and significantly differential lipids species in each subclass in positive ion modes (**B**) and negative ion modes (**C**).

Totally, 46 and 39 subclasses of lipids in live were detected in electron spray ionization positive and negative modes, i.e., ESI (+) and ESI (−), respectively, including 24 joint subclasses between them (Figure 5A). More details were shown in Figure 5B and Figure 5C, including 2064 lipid species in ESI (+) and 1154 lipid species in ESI (−) mode.

**Figure 6:**
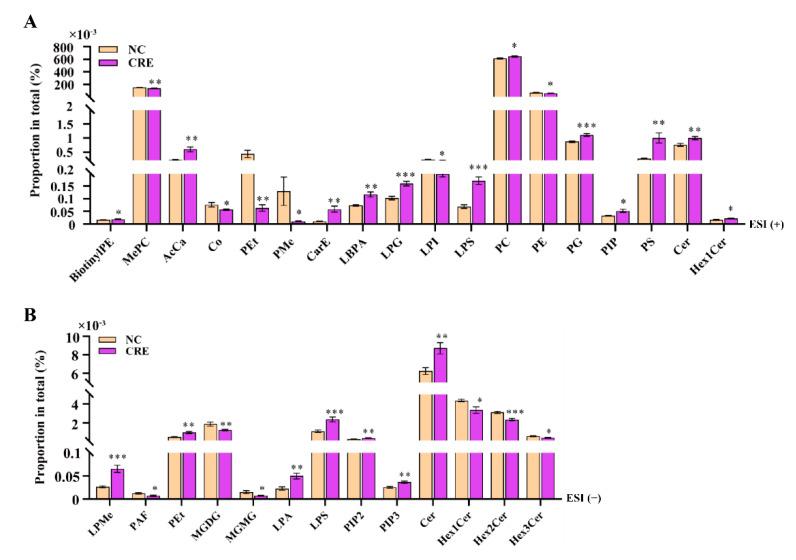
Paired comparison of the lipid composition in mice with or without CRE infection analyzed via the positive ion (**A**) and negative ion modes (**B**). Data are presented as mean ± SEM. * *p* < 0.05, ** *p* < 0.01, *** *p* < 0.001.

Further, the analyses of the differential subclasses of lipids upon CRE infection were performed. As shown in Figure 6A, twelve subclasses in ESI (+) mode including Cer, PC, PG, PIP, AcCa, BiotinylPE, CarE, Hex1Cer, LBPA, LPG, LPS, PG, PIP and PS increased significantly in the CRE group compared with the controls, while six subclasses of Co, PE, LPI, MePC, PEt and PMe significantly decreased. Meanwhile, seven subclasses in ESI (−) mode including Cer, LPA, LPMe, LPS, PEt, PIP2 and PIP3 significantly increased, while six subclasses including Hex3Cer, MGMG, Hex1Cer, Hex2Cer, MGDG and PAF significantly decreased upon CRE treatment (Figure 6B).

**Figure 7:**
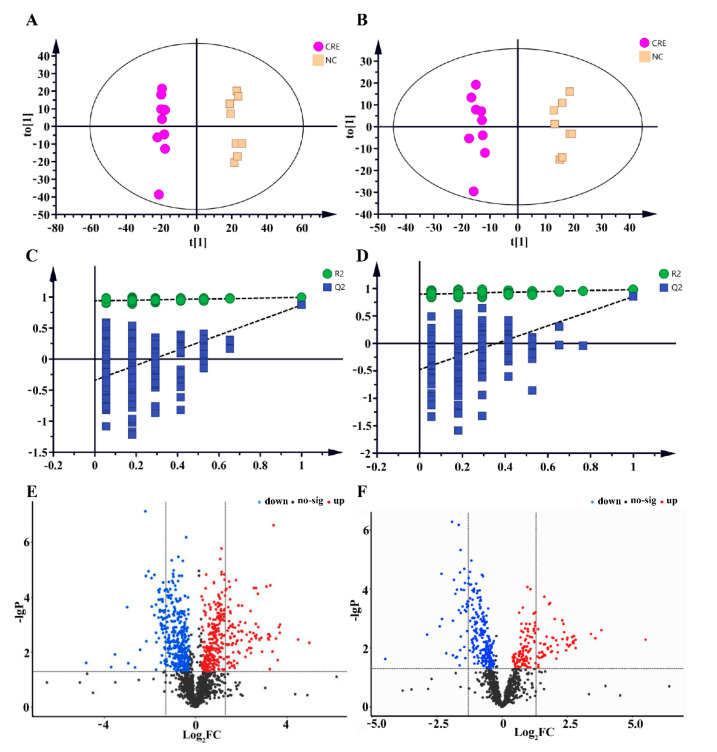
OPLS-DA score plot, permutation test and volcano plot for lipidomics in the positive ion (**A**,**C**,**E**) and negative ion modes (**B**,**D**,**F**). R2, explained variance; Q2, predictive ability of model. The red and blue dots in volcano plot represent the significantly up-regulated and down-regulated lipids species, respectively. FC, fold change.

**Figure 8:**
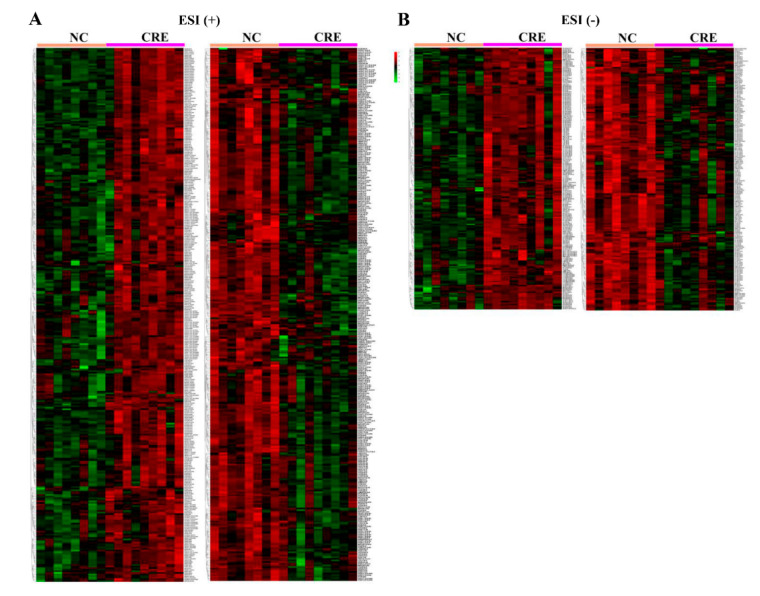
Heatmap showing the differential lipid species response to CRE exposure in the positive ion (**A**) and negative ion (**B**) modes.

To characterize the discriminatory lipid species response to CRE, a multivariable model of OPLS-DA was used. The mice in the CRE-infected group and the control group could be well-distinguished, with the model parameters of R^2^Y = 0.993 and Q^2^ = 0.871 in ESI (+) mode and R^2^Y = 0.981 and Q^2^ = 0.851 in ESI (−) mode, respectively (Figure 7A,B). Permutation test was used to verify the validity of the model and avoid the over-fitting. Figure 7C,D showed that the two OPLS-DA models were robust. Further univariate statistical assessments were performed by Student’s *t*-test and the calculation of the fold changes. Volcano plots were used to highlight the differentially expressed lipid species between groups (Figure 7E,F). A total of 386 differential lipids were found in the negative ion mode, of which 193 lipids were significantly enriched in the CRE group and 193 lipids were significantly depleted compared with the controls. A total of 664 differential lipids were found in the positive ion mode, of which 336 lipids were significantly enriched and 328 lipids depleted in the CRE group (Figure 8).

### 3.5. Association Analysis

The Spearman correlation analyses were performed to further identify the potential associations between the differential lipid species and lipid profiles of TC, TG, HDL-c and LDL-c in the liver. Results showed that a huge panel of lipid species were strongly correlated with the hepatic lipid profile, particularly TC and HDL-c. Exactly, in the positive ion mode of UPLC-QEMS, a total of 278 lipid species were positively and 208 were negatively correlated with TC, respectively. A total of 293 and 243 lipid species had positive and negative correlations with HDL-c. A total of 39 and 62 lipids were positively and negatively correlated with LDL-c. A total of six lipids were positively correlated with TG. Meanwhile, the levels of 203, 20, 212 and 54 lipid species detected in the negative ion mode were significantly correlated with the TC, TG, HDL-c and LDL-c, respectively (Table S3).

For the lipid species which had strong correlations with TC, TG, HDL-c and LDL-c, their associations with intestinal *Erysipelotrichaceae* family were analyzed. As shown in Table 1, the *Erysipelotrichaceae* family showed high correlation (|r| > 0.8 and *p* < 0.05) with 35 lipid species. Particularly, the levels of phospholipids including phosphatidylglycerol (42:2), phosphatidylglycerol (42:3), phosphatidylglycerol (38:5) and phosphatidylcholine (42:4), the sphingolipids of ceramide (d17:1/16:0) and ceramide (d18:1/16:0) and the glycolipids of diacylglycerol (20:2) had higher correlation coefficients of 0.90~0.97 with intestinal abundance of the *Erysipelotrichaceae* family.

### 3.6. Network Analysis

**Figure 9:**
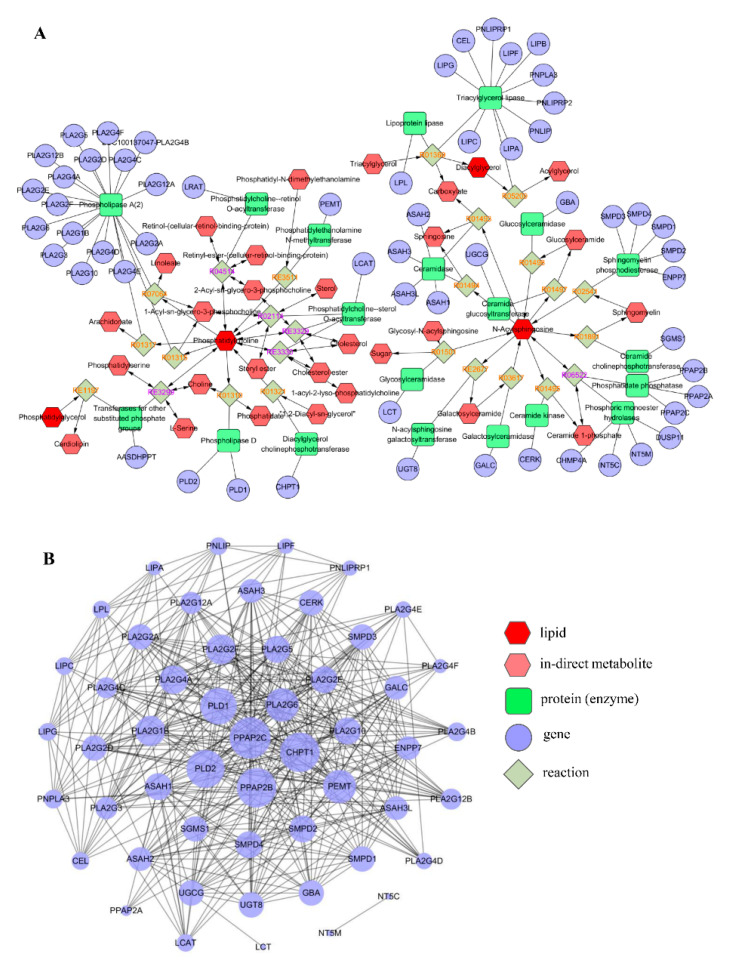
(**A**) The lipids–reaction–enzyme–gene interaction network. The red hexagon represents differential lipids; the pink hexagonal represents in-direct metabolites. The green square represents protein (enzyme). The purple circle represents genes that code for the protein. (**B**) Protein–protein interaction network on the targets potentially responsible for the altered lipid metabolism during CRE infection.

The lipid species which had strong correlations with the *Erysipelotrichaceae* family and lipid profile were mainly involved in four important classes of ceramide, phosphatidylglycerol, phosphatidylcholine and diacylglycerol. These lipids were adopted to construct the lipids–reaction–enzyme–gene interaction network, as shown in Figure 9A. Although it is challenging to understand how lipid composition is translated into function, totally, 58 targets were predicted to potentially associate with the abnormal lipid metabolism in mice infected with CRE. The protein–protein interaction was further analyzed, and the targets of PPAP2C, CHPT1, PPAP2B, PLD2 and PLD1 with higher degrees in the PPI network could be the key targets for preventing and treating the lipid metabolism disorder induced by CRE infection (Figure 9B).

## 4. Discussion

Systematic integration of multiple layers of omics datasets has been indicated to obtain more reliable results and reduce the false-positive risk [26,27], which can provide a basis for generating testable hypotheses and gaining mechanistic insights into the pathophysiology of multiple complex diseases in post-integration analyses [28,29]. In this study, the multi-omics technologies, including metagenomics, metabolomics and lipidomics, comprehensively characterized the alterations of gut microbiome and lipid metabolism occurring during the early infection of CRE in mice. Further integrated analyses revealed that the intestinal *Erysipelotrichaceae* family had a strong association with hepatic levels of total cholesterol, HDL-cholesterol and a large panel of lipid metabolites in mice with or without CRE exposure, which suggested the potential collusion effect of *Erysipelotrichaceae* during CRE infection. Lastly, a few targets were identified as potentially responsible for lipometabolic disturbances induced by CRE through network analysis.

Carbapenem resistance is more easily transferred horizontally and, therefore, spreads faster worldwide. The main mechanism for carbapenem antibiotics resistance in *Enterobacteriaceae* is the production of carbapenemase, a diverse family of β-lactamases [30] which worked by binding to the drug, breaking the amide bond of a four-membered azetidinone ring and preventing it from binding to the penicillin-binding protein of the bacterial cell wall [31]. Anyway, *Enterobacteriaceae* have alternative mechanisms for carbapenem resistance, including the production of other β-lactamases, porin loss and efflux pump overexpression [32], which block the penetration of the antibiotic within the bacterial cell. Cefiderocol, a recently emerging antibiotic with a unique chemical structure [33], exhibits excellent in vitro activity against many clinically relevant Gram-negative pathogens, including multidrug-resistant strains [34]. There is increasing evidence that cefoxiridol is well-suited to help address the growing number of infections caused by carbapenem-resistant and multidrug-resistant Gram-negative bacilli, including broad-spectrum β-lactamases and carbapenemase-producing strains [35].

Diverse roles of the gut microbiota in human health and disease have been recognized [36,37]. The 16S ribosomal RNA gene (16S) sequencing is a culture-free technique to identify the composition of intestinal microbial communities [38], aiming to look for correlations between the microbiota and disease or phenotype, to promote its application in exploring the microbial diversity of functional and pathogenic microorganisms and their interactions in biotechnology processes [39]. These culture-independent and reference-free approaches have proved to be successful strategies for species discovery and characterization [40,41]. Lots of studies have addressed the effect of antibiotic administration on the intestinal microbiota using sequencing technologies [42], revealing the ecological disturbances in the microbiota after antibiotic administration, especially for specific members of the bacterial community that are susceptible or resistant to antibiotics [43]. This post-antibiotic dysbiosis is usually characterized by a loss of diversity, a loss of certain important taxa, shifts in metabolic capacity and reduced colonization resistance against invading pathogens [44].

Infection altered the composition and diversity of gut microbiome, resulting in gut dysbiosis [45]. The family *Erysipelotrichaceae* has been reportedly linked to the host’s immune [46], which was also identified as a harmful bacterium due to the proinflammatory effect, and associated with elevated serum cholesterol levels [47]. Consistently, we found that the intestinal *Erysipelotrichaceae* family was strikingly increased upon CRE infection compared with controls and positively correlated with hepatic TC levels. The integrated analysis based on gut and metabolomics further showed that intestinal abundance of *Erysipelotrichaceae* and serum level of 24,25-dihydrolanosterol had a significantly positive association. The 24,25-dihydrolanosterol is an important cholesterol intermediate and is involved in the biosynthesis of steroids [48,49]. HDL-c mediates reverse cholesterol transport. In this study, hepatic levels of HDL-c unexpectedly increased in mice infected with CRE. Previous studies indicated that HDL-c has potent anti-inflammatory properties that may be critical for protection against infection [50]. The molecular mechanisms of how HDL-c can modulate inflammation is an interesting issue to be explored further.

Bacterial pathogens can recruit and use the lipids of a host and can hijack host lipid metabolism that facilitates the persistence of pathogens in the host [51]. For example, the survival of *Chlamydia* requires lipids from host cells and the absorption of sphingolipids and cholesterol from the host cells [52]. *M. tuberculosis* can alter the host lipid metabolism to create an environment that allows these intracellular pathogens to survive [53]. The invasion of exogenous pathogens can cause changes in enzymes and lipids that affect specific reactions [54]. *Pseudomonas aeruginosa* increased the enzymatic activity of the acid sphingomyelinase of macrophages, causing ceramide binding on the raft to activate the organism’s defenses [55]. Viral infection could induce the changes in the expression of cholesterol metabolic enzymes and metabolites in host cells, and the cholesterol metabolism regulated the antiviral response of host cells [56].

The liver, as the central organ in whole-body metabolism such as lipids, is the major source of fatty acid synthesis, as well as the lipoproteins released into the blood [57,58,59]. Our high-coverage lipidomic analysis showed that a huge panel of lipid species were significantly differential upon CRE infection. Further integrated analysis identified several lipid subclasses, belonging to sphingolipids, phospholipids and glycerolipids, that were significantly correlated with hepatic TC, HDL-c and the intestinal *Erysipelotrichaceae* family. Previous studies have shown that sphingolipid metabolism played a key role in the regulation of inflammatory signaling pathways [60], including bacterial pathogen infection, B cell activation and release of cytokines during infection [61]. Sphingolipids could also affect inflammation-related diseases by inhibiting intestinal lipid absorption [62,63], altering the intestinal microflora [64] and activating anti-inflammatory nuclear receptors [65,66]. Mammalian cell membranes primarily consist of phosphatidylcholine and cholesterol, while bacterial cell membranes are rich in amphoteric phosphatidylethanolamine, anionic phosphatidylglycerol and polyanionic cardiolipin. Pathogens can adapt to their biological sites by changing the composition of the membrane in order to evade the immune mechanisms of the antimicrobial substances and the host [67,68,69]. Triglycerides are located in adipocyte lipid droplets with vesicular transport and cell signal transduction functions, and it is the key to maintaining lipid balance [70].

The identification of the potential targets in this study could lead to a deeper understanding of the lipometabolic disturbance occurring during CRE infection. Phospholipid phosphatase of PPAP2C and PPAP2B participated in the ceramide metabolic process [71]. Cholinephosphotransferase families of CHPT1 and phosphatidylethanolamine N-methyltransferase (PEMT) were found to participate in the phosphatidylcholine biosynthetic process [72]. Phospholipase families of PLD2 and PLD1 were determined as targets for dyslipidemia [73] and can activate MAPK [74]. Cytosolic phospholipase families of PLA2G6, PLA2G1B, PLA2G2F and PLA2G4A played a major role in the remodeling of membrane lipids and the biosynthesis of lipid mediators of the inflammatory response [75]. Sphingomyelin phosphodiesterase families, such as SMPD2 and SMPD4, were associated with the internalization of pathogens, intracellular activation of signaling pathways, induction of apoptosis in infected cells and release of cytokines [76]. Several important metabolic pathways, including arachidonic acid metabolism, glycerophospholipid metabolism, glycosphingolipid metabolism and linoleate metabolism, were found through our metabolites–reaction–enzyme–gene network analysis. Previous studies have shown that the metabolic pathways of linoleic acid and arachidonic acid were up-regulated in the *Mycoplasma gallisepticum* and *Escherichia coli* co-infection model [77].

This study had some strengths and weaknesses. To the best of our knowledge, we firstly reported the potential association of the intestinal *Erysipelotrichaceae* family with hepatic lipid metabolism upon CRE infection. The integration of multi-omics analyses provided a novel insight to reveal the molecular characteristics of CRE infection. However, the mechanisms in which CRE infection affects commensal microbiota and their interplay within the host’s lipid metabolism need to be further studied. Second, adipose tissue plays a central role in systemic metabolic homeostasis. The adipose tissue morphology and the expressions or activities of the vital proteins, such as lipases, were not analyzed in this study, and the weights of brown adipose tissue were not measured. A recent study explored the response of adipocytes to bacterial infection and found that the expression of genes involved in fat metabolism decreased after infection, and the genes related to immune function and cytokine receptor genes were up-regulated, which indicates that the function of adipocytes during infection has changed significantly from lipid metabolism to host defense [78]. Therefore, the metabolomic and lipidomic analyses of adipose tissue upon CRE infection demand exploration in the future.

## 5. Conclusions

This pilot study provided a novel insight into CRE infection by a system biology strategy. Hepatic lipid accumulation and the systemic disturbance of gut microbiota were revealed during the early infection of CRE. Metabolomics and lipidomics comprehensively characterized the alterations of circulating metabolites related to lipids metabolism and hepatic lipids compositions response to CRE exposure. The increased intestinal colonization of the *Erysipelotrichaceae* family was strongly associated with the alterations of TC, HDL-c and a panel of lipid species, particularly those belonging to ceramide, phosphatidylglycerol, phosphatidylcholine and diacylglycerol. The integrated multi-omics study highlighted the interplay of commensals and pathogens for host’s lipid metabolism, which may lead to new therapeutic approaches against infectious diseases in the future. Further studies are needed to explain how host–microbiota–pathogen interactions favorably or negatively influence host survival during CRE infection.

## Figures and Tables

**Table 1:** Correlation analyses showing the associations of significantly differential lipid species with intestinal *Erysipelotrichaceae* family and hepatic total cholesterol and HDL-cholesterol (*p* < 0.05, |r|>0.8).

NO.	Class	Species	MODE	Adduct	CalcMz	Formula	VIP	P	FDR	FC	*Ery*	Correlation (r, *p*)			
												TG	TC	HDL-c	LDL-c
1	Sphingolipids	Cer (d17:1/16:0)	ESI (+)	[M+H-H_2_O]^+^	506.4932	C_33_H_64_O_2_N	1.48	1.45 × 10^−3^	2.3 × 10^−3^	1.87	0.93 ***	ns	0.83 ***	0.86 ***	0.6 *
2	Sphingolipids	Cer (d18:1/16:0)	ESI (−)	[M+HCOO]^−^	582.5103	C_35_H_68_O_5_N	1.81	2.87 × 10^−5^	2.52 × 10^−4^	2.04	0.9 ***	ns	−0.49 *	ns	ns
3	Sphingolipids	Cer (d18:1/16:0)	ESI (+)	[M+H-H_2_O]^+^	520.5088	C_34_H_66_O_2_N	1.79	1.49 × 10^−5^	1.74 × 10^−4^	2.19	0.87 **	ns	0.77 ***	0.81 ***	ns
4	Sphingolipids	Cer (d18:1/17:0)	ESI (+)	[M+H-H_2_O]^+^	534.5245	C_35_H_68_O_2_N	1.62	2.72 × 10^−4^	8.77 × 10^−4^	2.41	0.85 **	ns	0.76 ***	0.8 ***	ns
5	Sphingolipids	Cer (d18:2/16:0)	ESI (+)	[M+H]^+^	536.5037	C_34_H_66_O_3_N	1.57	6.1 × 10^−4^	1.08 × 10^−3^	2.04	0.83 **	ns	0.73 ***	0.81 ***	0.54 *
6	Sphingolipids	Cer (t17:0/16:0)	ESI (+)	[M+H-H_2_O]^+^	524.5037	C_33_H_66_O_3_N	1.63	2.76 × 10^−4^	8.77 × 10^−4^	2.16	0.88 **	ns	0.83 ***	0.86 ***	0.6 *
7	Sphingolipids	Cer (t18:1/16:0)	ESI (+)	[M+H-H_2_O]^+^	536.5037	C_34_H_66_O_3_N	1.58	5.56 × 10^−4^	1.08 × 10^−3^	2.06	0.83 **	ns	0.74 ***	0.81 ***	0.53 *
8	Sphingolipids	SM (d33:1)	ESI (+)	[M+H]^+^	689.5592	C_38_H_78_O_6_N_2_P	1.60	2.78 × 10^−4^	8.77 × 10^−4^	1.57	0.88 **	ns	0.69 **	0.73 ***	ns
9	Phospholipids	PG (40:8)	ESI (+)	[M+Na]^+^	841.499	C_46_H_75_O_10_PNa	1.41	2.03 × 10^−3^	2.54 × 10^−3^	1.62	0.82 **	ns	0.61 **	0.69 **	ns
10	Phospholipids	PG (42:2)	ESI (+)	[M+NH_4_]^+^	876.6688	C_48_H_95_O_10_NP	1.78	1.2 × 10^−5^	1.74 × 10^−4^	2.21	0.9 ***	ns	0.8 ***	0.87 ***	0.59 *
11	Phospholipids	PG (42:3)	ESI (+)	[M+NH_4_]^+^	874.6532	C_48_H_93_O_10_NP	1.61	3.01 × 10^−4^	8.77 × 10^−4^	2.38	0.92 ***	ns	0.74 ***	0.82 ***	ns
12	Phospholipids	PC (32:2)	ESI (+)	[M+H]^+^	730.5381	C_40_H_77_O_8_NP	1.60	4.48 × 10^−4^	1.04 × 10^−3^	0.29	−0.82**	ns	−0.67 **	−0.73 ***	ns
13	Phospholipids	PC (34:2e)	ESI (+)	[M+H]^+^	744.5902	C_42_H_83_O_7_NP	1.53	6.48 × 10^−4^	1.08 × 10^−3^	1.83	0.89 **	ns	0.73 ***	0.73 ***	ns
14	Phospholipids	PC (36:3)	ESI (+)	[M+H]^+^	784.5851	C_44_H_83_O_8_NP	1.45	1.77 × 10^−3^	2.54 × 10^−3^	4.13	0.82 **	ns	0.59 *	0.65 **	0.27 ns
15	Phospholipids	PC (42:4)	ESI (+)	[M+H]^+^	866.6633	C_50_H_93_O_8_NP	1.12	2.19 × 10^−2^	2.39 × 10^−2^	1.29	0.97 ***	ns	0.5 *	0.62 **	ns
16	Phospholipids	LPC (24:2)	ESI (+)	[M+H]^+^	604.4337	C_32_H_63_O_7_NP	1.06	2.95 × 10^−2^	2.95 × 10^−2^	1.32	0.88 **	ns	0.62 **	0.55 *	ns
17	Phospholipids	MePC (36:7)	ESI (+)	[M+Na]^+^	812.5201	C_45_H_76_O_8_NPNa	1.42	3.91 × 10^−3^	4.56 × 10^−3^	0.77	−0.88 **	ns	−0.68 **	−0.66 **	ns
18	Phospholipids	PG (18:0/18:2)	ESI (+)	[M+H]^+^	775.5484	C_42_H_80_O_10_P	1.37	3.62 × 10^−3^	4.37 × 10^−3^	1.94	0.85 **	ns	0.7 **	0.72 **	ns
19	Phospholipids	PG (18:1/18:2)	ESI (+)	[M+H]^+^	773.5327	C_42_H_78_O_10_P	1.71	5.52 × 10^−5^	3.82 × 10^−4^	2.49	0.85 **	ns	0.75 ***	0.82 ***	ns
20	Phospholipids	PG (38:5)	ESI (+)	[M+NH_4_]^+^	814.5593	C_44_H_81_O_10_NP	1.43	1.91 × 10^−3^	2.54 × 10^−3^	1.76	0.93 ***	ns	0.75 ***	0.78 ***	0.51 *
21	Phospholipids	LPG (18:2)	ESI (+)	[M+Na]^+^	531.2693	C_24_H_45_O_9_PNa	1.37	7.03 × 10^−3^	7.93 × 10^−3^	2.08	0.87 **	ns	0.77 ***	0.86 ***	0.55 *
22	Phospholipids	PS (42:0)	ESI (+)	[M+H]^+^	876.6688	C_48_H_95_O_10_NP	1.57	4.13 × 10^−4^	1.03 × 10^−3^	2.27	0.9 ***	ns	−0.52 *	ns	ns
23	Phospholipids	MLCL (62:1)	ESI (−)	[M-2H]^−^	653.4657	C_71_H_136_O_16_P_2_	1.40	2.01 × 10^−3^	2.54 × 10^−3^	1.38	0.85 **	−0.49*	−0.5 *	−0.37 ns	ns
24	Phospholipids	PIP (52:3)	ESI (+)	[M+Na]^+^	1187.747	C_61_H_114_O_16_P_2_Na	1.14	2.56 × 10^−2^	2.64 × 10^−2^	1.57	0.82 **	ns	0.54 *	0.63 **	ns
25	Phospholipids	PIP2 (18:1/20:4)	ESI (−)	[M-H]^−^	1043.467	C_47_H_82_O_19_P_3_	1.65	3.28 × 10^−4^	8.84 × 10^−4^	2.12	0.87 **	ns	0.8 ***	0.87 ***	0.58 *
26	Glycerolipids	DG (20:2)	ESI (+)	[M+NH_4_]^+^	414.3214	C_23_H_44_O_5_N	1.94	2.42 × 10^−7^	8.45 × 10^−6^	10.69	0.92 ***	ns	0.77 ***	0.82 ***	0.55 *
27	Glycerolipids	DG (34:1e)	ESI (+)	[M+Na]^+^	603.5323	C_37_H_72_O_4_Na	1.70	8.05 × 10^−5^	3.82 × 10^−4^	2.01	0.82 **	ns	0.66 **	0.7 **	ns
28	Glycerolipids	DG (36:4e)	ESI (+)	[M+H]^+^	603.5347	C_39_H_71_O_4_	1.70	8.05 × 10^−5^	3.82 × 10^−4^	2.01	0.82 **	ns	0.66 **	0.7 **	ns
29	Glycerolipids	DG (38:6e)	ESI (+)	[M+H]^+^	627.5347	C_41_H_71_O_4_	1.57	6.54 × 10^−4^	1.08 × 10^−3^	2.24	0.87 **	ns	0.66 **	0.78 ***	ns
30	Glycerolipids	TG (17:0/11:2/11:2)	ESI (+)	[M+NH_4_]^+^	690.5667	C_42_H_76_O_6_N	1.53	5.94 × 10^−4^	1.08 × 10^−3^	1.54	0.83 **	ns	0.72 **	0.72 **	ns
31	Glycerolipids	TG (22:6/12:4/14:4)	ESI (+)	[M+Na]^+^	801.5065	C_51_H_70_O_6_Na	1.68	8.72 × 10^−5^	3.82 × 10^−4^	2.44	0.83 **	ns	0.82 ***	0.83 ***	0.56 *
32	Fatty acyl and others	AEA (18:2)	ESI (+)	[M+H]^+^	324.2897	C_20_H_38_O_2_N	1.14	2.3 × 10^−2^	2.44 × 10^−2^	2.77	0.82 **	ns	0.92 ***	0.91 ***	0.69 **
33	Fatty acyl and othes	AEA (20:3)	ESI (+)	[M+H]^+^	350.3054	C_22_H_40_O_2_N	1.47	1.96 × 10^−3^	2.54 × 10^−3^	1.56	0.87 **	0.52*	0.89 ***	0.82 ***	0.71 **
34	Fatty acyl and others	AcCa (22:1)	ESI (+)	[M+H]^+^	482.4204	C_29_H_56_O_4_N	1.54	5.2 × 10^−4^	1.08 × 10^−3^	2.37	0.83 **	ns	0.64 **	0.72 **	ns
35	Fatty acyl and others	PEt (18:1/22:6)	ESI (−)	[M-H]^−^	773.5127	C_45_H_74_O_8_P	1.50	1.78 × 10^−3^	2.54 × 10^−3^	1.89	0.9 ***	−0.49*	ns	ns	ns

VIP was obtained from OPLS-DA with a threshold of 1.0. *p* values were calculated from Student’s *t*-test; FDR was obtained from the adjusted *p* values based on the Benjamini and Hochberg method; FC was calculated from the arithmetic mean values of CRE-treated group to control. * *p* < 0.05, ** *p* < 0.01, *** *p* < 0.001. Abbreviations: *Ery*, *Erysipelotrichaceae*; Cer, ceramide; PG, phosphatidylglycerol; PC, phosphatidylcholine; DG, diacylglycerol; TG, triacylglycerol; AEA, N-Acylethanolamine; AcCa, Acyl Carnitine; LPC, lysophosphatidylcholine; LPG, lysophosphatidylglycerol; MePC, Methyl phosphatidylcholine; MLCL, Mono-lyso cardiolipin; PEt, Phosphatidylethanol; PIP, Phosphatidylinositol phosphate; P1P2, Phosphatidylinositol diphosphate; PS, phosphatidylserine; SM, sphingomyelins.

## Data Availability

All data generated or analyzed during this study are included in this published article and its Supplementary Materials. Raw data of all quantitatively analyzed experiments are available from the corresponding author on reasonable request.

## Supplementary Materials

The following supporting information can be downloaded at: https://www.mdpi.com/article/10.3390/metabo12100892/s1, Table S1: Metabolomics analysis showing 74 significantly different metabolites in serum during early infection of CRE. Table S2: Metabolomics analysis showing 129 significantly different metabolites in feces during early infection of CRE. Table S3: Correlation analyses showing the associations of significantly differential lipid species with TC, TG, HDL-c and LDL-c levels in liver. * *p* < 0.05, ** *p* < 0.01, *** *p* < 0.001.
